# The β-fibrinogen gene 455G/A polymorphism associated with cardioembolic stroke in atrial fibrillation with low CHA_2_DS_2_-VaSc score

**DOI:** 10.1038/s41598-017-17537-1

**Published:** 2017-12-13

**Authors:** Xiaofeng Hu, Junjun Wang, Yaguo Li, Jiong Wu, Song Qiao, Shanhu Xu, Jun Huang, Linhui Chen

**Affiliations:** 1 Department of Cardiology, Zhejiang Hospital, Hangzhou, 310013 Zhejiang Province China; 2 Department of Neurology, Zhejiang Hospital, Hangzhou, 310013 Zhejiang Province China; 3 Institute of Biostatistics, School of Life Sciences Fudan University, Shanghai, 200082 China

## Abstract

Previous work has suggested that ischemic stroke (IS) may be more likely to occur in individuals with a genetic predisposition. In this study, we investigated the potential association of IS-relevant genetic risk factors with cardioembolic stroke (CES) in atrial fibrillation (AF) patients with low CHA2DS2-VaSc score. Genotyping was performed using the GenomeLab SNPstream genotyping platform for five IS-relevant SNPs (MMP-9 C1562T, *ALOX5AP* SG13S114A/T, *MTHFR* 677 C/T, *FGB* 455 G/A, and *eNOS* G298A) in 479 AF patients with CES and 580 age and sex-matched AF patients without CES. The multivariate analysis adjusted for potential confounders and demonstrated that *FGB* 455 G/A was independently associated with increased risk of CES in AF patients and the significance remained after Bonferroni correction in the additive, dominant, and recessive models with ORs of 1.548 (95% CI: 1.251–1.915, *P* = 0.001), 1.588 (95% CI: (1.226–2.057, *P* = 0.003), and 2.394 (95% CI: 1.357–4.223, *P* = 0.015), respectively. Plasma fibrinogen levels were significantly higher in patients with the A allele compared with patients with genotype of GG (3.29 ± 0.38 mg/dl vs. 2.87 ± 0.18 mg/dl, P < 0.001). We found for the first time that the A allele of *FGB* 455 G*/*A was a risk factor for CES in AF patients, probably by elevating the level of plasma fibrinogen.

## Introduction

Ischemic stroke (IS) is the most common type of stroke in China, resulting in a heavy socioeconomic burden. Cardioembolic stroke (CES) accounts for approximately one third of all IS and is considered one of the more preventable types of strokes^1^. Atrial fibrillation (AF) is the most frequent cause of CES, but this risk varies widely among AF patients and depends on the presence of various stroke risk factors^2^. To date, several clinical risk factors have been identified that contribute to the pathogenesis of IS in AF patients, including age, hypertension, diabetes mellitus, congestive heart failure, vascular disease, and female^3,4^. These clinical risk factors have been used to formulate stroke risk stratification schemes, such as CHA_2_DS_2_ and CHA_2_DS_2_-VaSc scores. Patients with CHA_2_DS_2_-VASc score ≥2 were defined as high risk and would be recommended to receive oral anticoagulation therapy. Patients with scores of 0 and 1 are defined as low risk, but a proportion of these patients suffer from CES. The risk of CES in AF patients with low CHA_2_DS_2_-VaSc score have often been underestimated, especially in Asian people^5–7^. The American guidelines suggest anti-platelets therapy may be recommended in patients with a CHA_2_DS_2_-VaSc score of 1^8^. It would be of great clinical significance to identify individuals at relatively high risk of CES with a low CHA_2_DS_2_-VaSc score of either 0 or 1.

Recent work has suggested that IS may be more likely to occur in individuals with a genetic predisposition^9^. Previously published reports demonstrated a relationship between the IS and functional variation evidenced by single nucleotide polymorphisms (SNPs) in the matrix metallopeptidas-9 gene (*MMP-9*)^10,11^, the arachidonate 5-lipoxygenase-activating protein gene (*ALOX5AP*)^12–14^, the methylene tetrahydrofolate reductase gene (*MTHFR*)^15^, the β-fibrinogen gene (*FGB*)^16,17^, and the endothelial nitric oxide synthase gene (*eNOS*)^18,19^. However, whether IS-relevant genetic factors convey a risk for CES in AF patients with low CHA_2_DS_2_-VASc score remains unknown. The identification of genes causally related to CES may provide a better understanding of CES pathogenesis and may potentially inform the development of therapies for the prevention of CES in AF patients with low CHA_2_DS_2_-VaSc score, thus there is a critical need to identify the genetic risk of CES in this population. Appropriate anticoagulation is the most significant factor that determines the occurrence of CES in AF patients. Therefore, our study subjects were restricted to patients with low CHA_2_DS_2_-VaSc score who received no anticoagulation therapy. The aim of this study was to identify genetic factors that can predict CES in non-valvular AF patients with a low CHA_2_DS_2_-VaSc score.

## Materials and Methods

### Study population

A total of 479 consecutive AF patients (score = 0 or 1) with an initial diagnosis of CES from the Second Affiliated Hospital of Zhejiang University were enrolled in the study. All patients were diagnosed at the hospital from January 2012 to December 2015. Control subjects (n = 580) were AF subjects who underwent a routine medical check-up in the outpatient clinic of the Department of Cardiology at the Second Affiliated Hospital of Zhejiang University during the same period. The controls were frequency-matched to cases on the basis of age and sex. The CHA_2_DS_2_-VASc score was calculated for each patient as follows: two points were assigned for a history of stroke or transient ischemic attack (TIA), or age ≥75 years; and 1 point was assigned for heart failure, hypertension, age 65–74 years, diabetes mellitus, vascular disease, and female sex^20^.

AF was diagnosed according to the 2014 AHA/ACC/HRS Guideline for the Management of Patients with Atrial Fibrillation^8^. AF was diagnosed by 12-lead electrocardiogram or 24 h dynamic electrocardiogram and only those patients with documented AF (>6 minutes) were included. Exclusion criteria are as follows: patients with history of cerebral ischemic events; receiving oral anticoagulation therapy; with severe hepatic or renal dysfunction; with congenital heart disease; with rheumatologic disorders; with organic valvular heart diseases; with infective endocarditis; with hyperthyroidism; or with tumors or severe infections.

At admission, data on patient characteristics, including age, gender, body mass index (BMI), the history of hypertension, diabetes mellitus, vascular disease, congestive heart failure, low-density lipoprotein cholesterol (LDL-C), high-density lipoprotein cholesterol (HDL-C), plasma fibrinogen level, platelet count, D-dimer, high sensitive C reaction protein (hs-CRP), left atrial diameter (LAD), left ventricle ejection fraction (LVEF), lifestyle (e.g., cigarette smoking and alcohol consumption), and antiplatelet therapy. Fibrinogen levels were measured in blood samples derived from peripheral venous punctures on the day of hospital admission. All participants were unrelated Han Chinese who were consecutively selected from the same geographic region. The protocol in this study conformed to the principles of the Declaration of Helsinki and was ratified by the Human Ethical Committee of the Second Affiliated Hospital of Zhejiang University. Informed consent was obtained from all subjects.

### CES diagnosis

CES was diagnosed according to the TOAST criteria and based on the clinical findings, neuroimaging data (cranial magnetic resonance imaging (MRI) and/or computed tomography (CT)), and results of diagnostic studies such as cardiac imaging (echocardiography), ECG, duplex imaging of extracranial arteries, and laboratory evaluation^21^. Unless other investigations (e.g. high-grade internal carotid artery stenosis) showed otherwise, all IS in AF patients were defined as CES.

### Selection of SNPs and genotyping

Five SNPs were selected based on positive associations in previous studies with a minor allele frequency (MAF) of >5% in the Chinese Han population (http://www.1000genomes.org//) and underlying biological plausibility. The following SNPs were determined: MMP-9 gene C1562T (rs#3918242), ALOX5AP gene SG13S114A/T (rs#10507391), MTHFR gene 677 C/T (rs#1801133), FGB gene 455 G/A (rs#1800790), and eNOS gene G894T (rs#1799983) (Table 1).

**Table 1:** Genomic characteristic of studied SNPs.

Gene	SNP	rs number	Locus	Major/minor	Variant class
*MMP-9*	C1562T	rs3918242	20q13	C/T	5′UTR
*ALOX5AP*	SG13S114A/T	rs10507391	13q12	A/T	Intronic
*MTHFR*	677 C/T	rs1801133	1p36	C/T	Exonic (Ala-Val)
*FGB*	455 G/A	rs1800790	4q31	G/A	5′UTR
*eNOS*	G894T	rs1799983	7q35–36	G/T	Exonic (Glu-Asp)

SNP: single nucleotide polymorphism; UTR: untranslated region; MMP-9: matrix metallopeptidas-9 gene; ALOX5AP: arachidonate 5-lipoxygenase-activating protein gene; MTHFR: methylene tetrahydrofolate reductase gene; FGB: the β-fibrinogen gene; and eNOS: endothelial nitric oxide synthase gene.

The genomic DNA was isolated from whole blood samples using the whole blood DNA kit (Tiangen Biotech, Beijing, China). The concentration of DNA was diluted to 20 ng/μl for working solutions and the isolated DNA was stored at −20 °C. SNP genotyping was conducted by Orchid BioSciences using the GenomeLab SNPstream genotyping platform (Beckman Statistical analyses) and SNPstream software suite. Two independent research assistants read the results with blindness of cases and controls. For quality control, distilled water was used as a negative control. Ambiguous genotyping results were verified by sequencing analysis.

### Statistical analyses

Mean ± SD, median, and interquartile were separately used to describe the continuous variables with normal and skewed distribution. Student’s t test, or Mann-Whitney U test were applied to compare demographic and clinical data between groups as appropriate. Categorical variables were represented by frequencies and percentages, and were compared using chi-squared tests. Allele case-control comparisons were analyzed by Pearson’s chi-square test or Fisher’s exact test. The Hardy-Weinberg equilibrium (HWE) was independently evaluated for each polymorphism. Logistic regression was performed to assess the association between the presence of a particular genotype and CES. The following analytical methods were used to compare the subjects from two groups: allelic frequency distribution of the two groups (allele A versus allele B, A as the major allele, B as the minor allele, this also applied to the following methods); additive model (BB versus AB versus AA); dominant model (AB + BB versus AA); and recessive model (BB versus AA + AB). All the genetic models of the minor allele were performed with or without adjustment for confounding risk factors. All odds ratios (ORs) were given with the 95% confidence interval (CI). Furthermore, the Bonferroni correction was used to define the effective number of independent marker loci. Statistical analyses were performed using SAS Version 9.1 (SAS Institute, Cary, North Carolina, USA). A two-sided *P* value < 0.05 was considered to be statistically significant.

### Data Availability

The datasets generated during and/or analysed during the current study are available from the corresponding author on reasonable request.

## Results

### Characteristics of the included subjects

A total of 1059 AF patients (479 CES patients and 580 controls) participated in the study. The age and sex of the participants from the two groups were matched (*P* = 0.834 and 0.498, respectively). Compared with the control group, patients with CES had significantly higher prevalence of hypertension (20.9% vs. 11.7%, *P* < 0.001), larger LAD (38.26 ± 4.90 mm vs. 38.54 ± 4.72 mm, *P* = 0.015), higher LDL-C level (3.08 ± 0.53 vs. 2.99 ± 0.55, P = 0.009), higher plasma fibrinogen level (3.07 ± 0.38 mg/dl vs. 3.00 ± 0.31 mg/dl, *P* = 0.003), higher hs-CRP level (3.92 ± 5.06 mg/l vs. 3.11 ± 5.26 mg/l, *P* = 0.011), and higher CHA_2_DS_2_-VaSc score (0.94 ± 0.24 vs. 0.87 ± 0.34, *P* < 0.001). There was no significant difference between the two groups for BMI, smoking, drinking, AF type, history of AF, diabetes mellitus, vascular disease, congestive heart failure, LVEF, HDL-C, platelet count, D-dimer, and antiplatelet therapy (Table 2).

**Table 2:** Characteristics of the participants.

Variable	AF patients with CES (n = 479)	AF patients without CES (n = 580)	*P*
Age (years)	65.18 ± 5.26	65.11 ± 5.67	0.834
Female, n (%)	53(11.1%)	72(12.4%)	0.498
BMI (kg/m^2^)	23.49 ± 2.85	23.33 ± 2.90	0.372
Smoking, n (%)	177(37.0%)	210(36.2%)	0.802
Drinking, n (%)	161(33.6%)	189(32.6%)	0.724
Non-paroxysmal AF, n (%)	232(48.4%)	275(47.4%)	0.741
History of AF (IQR, months)	33(26–36)	31(26–35.5)	0.376
Hypertension, n (%)	100(20.9%)	68(11.7%)	< 0.001
Diabetes mellitus, n (%)	29(6.1%)	32(5.5%)	0.709
Vascular disease, n (%)	19(3.8%)	18(3.3%)	0.671
Congestive heart failure, n (%)	8(1.6%)	8(1.3%)	0.699
CHA_2_DS_2_-VaSc score	0.94 ± 0.24	0.87 ± 0.34	< 0.001
0	29(6.0%)	77(13.3%)	
1	450(94.0%)	503(86.7%)	
LVEF (%)	60.77 ± 5.31	60.87 ± 5.10	0.751
LAD (mm)	38.26 ± 4.90	38.54 ± 4.72	0.015
LDL-C (mmol/l)	3.08 ± 0.53	2.99 ± 0.55	0.009
HDL-C (mmol/l)	1.36 ± 0.27	1.37 ± 0.32	0.559
Fibrigen (mg/dl)	3.07 ± 0.38	3.00 ± 0.31	0.003
Platelet count (10^9^/l)	205 ± 36	203 ± 35	0.488
D-dimer (mg/dl)	0.31 ± 0.07	0.30 ± 0.08	0.320
hs-CRP (mg/l)	3.92 ± 5.06	3.11 ± 5.26	0.011
Antiplatelet therapy, n (%)	226(47.2%)	267(46.0%)	0.710

AF: atrial fibrillation; CES: cardioembolic stroke; BMI: body mass index; LVEF: left ventricle ejection fraction; LAD: left atrial diameter; LDL-C: low-density lipoprotein cholesterol; HDL-C: high-density lipoprotein cholesterol; hs-CRP: high sensitivity C-reactive protein.

### Genotypic and allelic distributions of the five SNPs

The genotyping success rates of the five SNPs ranged from 99.6–100%. The veracity of the results was confirmed by direct sequencing of PCR products amplified from randomly selected samples. The direct sequencing results were consistent with all corresponding genotyping results. All genotypes were distributed in concordance with Hardy-Weinberg equilibrium (HWE) with a value of *P* > 0.05 in control group, minimizing the possibility of selection bias.

Of the five SNPs, significant differences in genotypic and allelic distribution were only identified for the *FGB* 455 G/A polymorphism between the CES and control groups (*P* = 0.008 and *P* < 0.001, respectively) (Table 3). There were more A allele carriers of the *FGB* 455 G/A polymorphism in CES group compared with the number in the control group (25.5% vs. 18.9%, *P* < 0.001).

**Table 3:** Association analysis of 5 genotyped SNPs of the *MMP9*, *ALOX5AP*, *MTHFR*, *FGB*, and *eNOS* genes with CES.

**Gene**	**SNP**	**Genotype**	**AF patients with CES (n** **=** **479)**		**AF patients without CES (n** **=** **580)**		***P***	***P***_**HWE**_
			**No**	**Frequency**	**No**	**Frequency**		
*MMP9*	C1562T	CC	293	61.5%	357	61.8%	0.963	0.921
		CT	160	33.5%	194	33.5%		
		TT	24	5.0%	27	4.7%		
		C:T		0.78:0.22		0.79:0.21	0.846	
*ALOX5AP*	SG13S114A/T	AA	147	30.7.2%	205	35.3%	0.116	0.500
		AT	256	53.4%	273	47.1%		
		TT	76	15.9%	102	17.6%		
		A:T		0.57:0.43		0.59:0.41	0.495	
*MTHFR*	677 C/T	CC	285	59.5%	321	55.5%	0.425	0.298
		CT	162	33.8%	213	36.9%		
		TT	32	6.7%	44	7.6%		
		C:T		0.76:0.24		0.74:0.26	0.195	
*FGB*	455 G/A	GG	271	56.6%	382	65.9%	0.001	0.929
		GA	172	35.9%	177	30.5%		
		AA	36	7.5%	21	3.6%		
		G:A		0.75:0.25		0.81:0.19	< 0.001	
*eNOS*	G894T	GG	338	70.6%	418	72.3%	0.437	0.058
		GT	129	26.9%	140	24.2%		
		TT	12	2.5%	20	3.5%		
		G:T		0.84:0.16		0.84:0.16	0.802	

AF: atrial fibrillation; CES: cardioembolic stroke; SNP: single nucleotide polymorphism; MMP-9: matrix metallopeptidas-9 gene; ALOX5AP: arachidonate 5-lipoxygenase-activating protein gene; MTHFR: methylene tetrahydrofolate reductase gene; FGB: the β-fibrinogen gene; and eNOS: endothelial nitric oxide synthase gene.

### Association between *FGB* 455 G/A polymorphism and risk of CES in AF patients

In univariate analysis, we detected significant association between *FGB* 455 G/A and risk of CES in the additive model (OR = 1.452, 95% CI: 1.183–1.783, *P* < 0.001), dominant model (OR = 1.481, 95% CI: 1.154–1.900, *P* = 0.002), and recessive model (OR = 2.163 95% CI: 1.245–3.758, *P* = 0.006). Similar results were obtained after adjusting for confounding factors such as age, sex, BMI, smoking, drinking, hypertension, diabetes mellitus, heart failure, vascular disease, LAD, AF type, LDL, and CRP. After Bonferroni correction, the significance remained in the additive, dominant, and recessive models with ORs of 1.548 (95% CI: 1.251–1.915, *P*_Bonferroni_ = 0.001), 1.588 (95% CI: (1.226–2.057, *P*_Bonferroni_ = 0.003), and 2.394 (95% CI: 1.357–4.223, *P*_Bonferroni_ = 0.015), respectively (Table 4).

**Table 4:** Association between *FGB* 455 G/A polymorphism and risk of CES in AF patients.

Genetic model	Crude OR (95% CI)^*^	*P*	Adjusted OR (95% CI)^**^	Adjusted P	*P*_Bonferroni_
Additive model	1.452(1.183,1.783)	<0.001	1.548(1.251–1.915)	<0.001	0.001
Dominant model	1.481(1.154–1.900)	0.002	1.588(1.226–2.057)	<0.001	0.003
Recessive model	2.163(1.245–3.758)	0.006	2.394(1.357–4.223)	0.003	0.015

^*^Crude ORs were calculate by univariate logistic regression analysis. ^**^Adjusted ORs were obtained from multivariate logistic regression additionally adjusted by age, sex, BMI, smoking, drinking, hypertension, diabetes mellitus, heart failure, vascular disease, LAD, AF type, LDL, and CRP. Additive model (AA vs. GA vs. GG). Dominant model (AA + GA vs. GG). Recessive model (AA vs. GA + GG). OR: odds ratio; CI: confidence interval.

### *FGB* 455 G/A polymorphism and plasma fibrinogen levels

**Figure 1:**
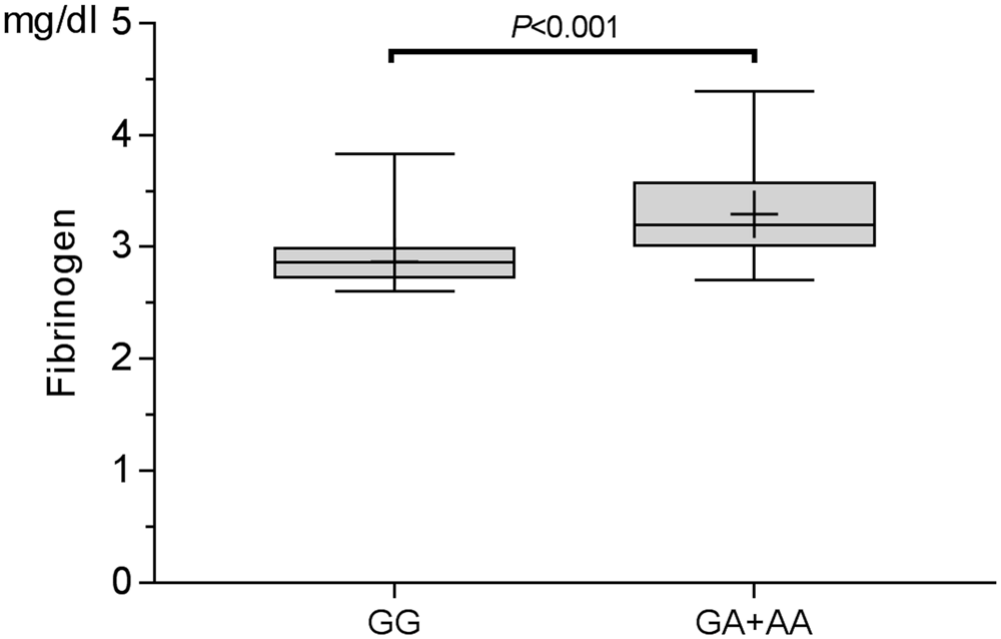
Plasma fibrinogen levels in patients with GA + AA genotype compared with those with GG genotype. Plasma fibrinogen level was significantly higher in GA + AA genotype (3.29 ± 0.38 mg/dl) compared with GG genotype (3.29 ± 0.38 mg/dl vs. 2.87 ± 0.18 mg/dl, *P* < 0.001, unpaired t test). The box represents the limits of the second and third quartiles; the horizontal band is the median, and the small cross is the mean. Whiskers represent minimum and max values.

Plasma fibrinogen level for the *FGB* gene GG genotype, GA genotype, and AA genotype were 2.87 ± 0.18 mg/dl, 3.22 ± 0.33 mg/dl, and 3.73 ± 0.43 mg/dl, respectively. There was a trend towards increasing plasma fibrinogen between *FGB* 455 G/A genotype. The plasma fibrinogen level was significantly higher in GA + AA genotype (3.29 ± 0.38 mg/dl) compared with the level in the GG genotype group (2.87 ± 0.18 mg/dl, *P* < 0.001, unpaired t test; Fig. 1).

## Discussion

In this contemporary case-control study, we investigated the association of five common genetic variants with CES in AF patients based on a Chinese Han population. We found that the *FGB* 455 G/A polymorphism was independently associated with increased risk of CES in AF patients with low CHA_2_DS_2_-VASc score. Our current study supports an important role of genetic predisposition in the pathogenesis of CES in AF patients.

Earlier studies explored relationships between the *FGB* 455 G/A polymorphism and IS in different populations. Kessler *et al*. reported that the AA genotype of the *FGB* 455 G/A polymorphism occurred significantly more frequently in patients with large vessel infarcts^16^. Nishiuma *et al*. found that the A allele of the 455 G/A polymorphism was an independent risk factor of IS in hypertensive patients in a Japanese population^22^. Martiskainen *et al*. demonstrated that the A allele of this polymorphism may predispose people to multiple lacunar infarcts^17^. Similarly, Zhang *et al*. reported that this polymorphism appears to be a genetic risk factor for IS in the Chinese population^23^. Several large meta-analyses confirmed the association of the *FGB* 455 G/A polymorphism with IS in Chinese or Asian population^24–26^. Although this polymorphism has been extensively studied for its association with IS, no study has demonstrated its genetic impact on the pathogenesis of CES in AF patients. To our knowledge, this is the first study demonstrating that a functional SNP in *FGB* is linked to an increased risk of CES in AF patients.

The exact mechanism by which the *FGB* 455 G/A polymorphism may affect CES pathology remains unknown. Promoter elements have primary roles in regulating gene transcription. A promoter variant may alter transcription factor binding sites or transcription initiation rates^27^. Experimental studies have reported that the *FGB* 455 G/A polymorphism has a substantial stimulatory effect on both the basal and stimulated rate of transcription of the *FGB* gene, and the A allele was associated with a significant increase in promoter activity^28,29^. Based on epidemiological and biochemical studies, the *FGB* 455 G/A polymorphism is one of the strongest genetic variations associated with an increase in plasma fibrinogen^30–32^. Being homozygous for the A allele is associated with increased levels of fibrinogen of approximately 0.30 g/l compared with G allele homozygotes^33^. Consistent with previous studies, we report here that the patients with presence of A allele of FGB 455 G/A polymorphism had a significantly higher fibrinogen level. Fibrinogen is an important component of the coagulation cascade and a major determinant of platelet aggregation and blood viscosity^34^. Elevated fibrinogen levels induce a state of hypercoagulability that may contribute to the progression of thrombosis^35,36^. Consistent with this, animal studies have demonstrated that administration of fibrinogen in increasing doses enhances experimentally-induced thrombosis and increases the number of emboli and the duration of embolization^37^. Additionally, fibrinogen is a key component of inflammation, triggering a variety of inflammatory processes which could cause fluctuations of thrombus plaque and result in IS^38^. These effects collectively could lead to hemorheological impairments, thus contributing to CES.

It is important to note that the functional effect of the *FGB* 455 G/A polymorphism remains controversial and other SNPs in the fibrinogen gene have also been implicated in causing higher fibrinogen concentrations. Haplotype analyses have shown that other SNPs in the *FGB* promoter region are functional SNPs but the 455 G/A may not be functional^39^. Additionally, the association of the 455 G/A polymorphism with higher fibrinogen concentrations may actually be due to linkage disequilibrium between the 455 G/A polymorphism and other causal polymorphisms^39^. Additionally, several studies have failed to provide evidence to support the association between 455 G/A polymorphism and thrombotic events^40–42^. We propose that the discrepancies in the results between studies may result from differences in ethnic background, sample size, and other factors.

The CHA_2_DS_2_-VASc score is a well validated and widely used clinical risk prediction tool for IS in non-valvular AF, however, so far such division of IS patients related to AF has never been performed according to the genetic risk factors. The risk of IS in Asian people is quite different from that in Western people, especially in patients with a low CHA_2_DS_2_-VaSc score of either 0 or 1^7^. Our results provided evidence of the significance of *FGB* variants in the future genotype-specific risk stratification of CES in AF patients with low CHA_2_DS_2_-VaSc score, which should allow improved decision support for the care of these relatively high risk patients.

This study has several limitations that should be acknowledged. First, this study is a cross-sectional study and is therefore subject to the limitations of this type of clinical analysis. The conclusions may be more precise if it is prospectively validated in an external large cohort of AF populations. Second, limited sample size needs to be considered as a potential source of heterogeneity, since studies with large sample size are more robust to random error and tend to reach a more objective result. Therefore, Bonferroni correction was performed in our study to show credibility of genetic association. Third, we cannot formally exclude the possibility that there are other loci in the *FGB* gene (or in other genes that have not yet been identified) that are linked with the *FGB* 455 G/A polymorphism and are the true cause of effects on CES in AF patients.

In conclusion, our study provides evidence for a potential association of *FGB* 455 G/A polymorphism with increased risk of CES in AF patients with low CHA_2_DS_2_-VaSc score. However, it is unclear whether this finding will be reproduced in other populations. Therefore, future well-designed large-scale studies in larger populations are still warranted to validate this finding.
